# *Ex vivo *promoter analysis of antiviral heat shock cognate 70B gene in *Anopheles gambiae*

**DOI:** 10.1186/1743-422X-5-136

**Published:** 2008-11-05

**Authors:** Seokyoung Kang, Cheolho Sim, Brian D Byrd, Frank H Collins, Young S Hong

**Affiliations:** 1 Department of Tropical Medicine, School of Public Health and Tropical Medicine, Tulane University, New Orleans, Louisiana 70112, USA; 2 Department of Entomology, the Ohio State University, Columbus, Ohio 43210, USA; 3 Environmental Health Sciences, Western Carolina University, Cullowhee, NC 28723, USA; 4 The Center for Global Health and Infectious Diseases, University of Notre Dame, Notre Dame, IN 46556, USA

## Abstract

**Background:**

The *Anopheles gambiae *heat shock cognate gene (*hsc70B*) encodes a constitutively expressed protein in the *hsp70 *family and it functions as a molecular chaperone for protein folding. However, the expression of *hsc70B *can be further induced by certain stimuli such as heat shock and infection. We previously demonstrated that the *An. gambiae hsc70B *is induced during o'nyong-nyong virus (ONNV) infection and subsequently suppresses ONNV replication in the mosquito. To further characterize the inducibility of *hsc70B *by ONNV infection in *An. gambiae*, we cloned a 2.6-kb region immediately 5' upstream of the starting codon of *hsc70B *into a luciferase reporter vector (pGL3-Basic), and studied its promoter activity in transfected Vero cells during infection with o'nyong-nyong, West Nile and La Crosse viruses.

**Results:**

Serial deletion analysis of the *hsc70B *upstream sequence revealed that the putative promoter is likely located in a region 1615–2150 bp upstream of the *hsc70B *starting codon. Sequence analysis of this region revealed transcriptional regulatory elements for heat shock element-binding protein (HSE-bind), nuclear factor κB (NF-κB), dorsal (Dl) and fushi-tarazu (Ftz). Arbovirus infection, regardless of virus type, significantly increased the *hsc70B *promoter activity in transfected Vero cells.

**Conclusion:**

Our results further validate the transcriptional activation of *hsc70B *during arbovirus infection and support the role of specific putative regulatory elements. Induction by three taxonomically distinct arboviruses suggests that the HSC70B protein may be expressed to cope with cellular stress imposed during infection.

## Introduction

The *Anopheles gambiae *mosquito is the principle vector of the malaria parasite *Plasmodium falciparum *in sub-Saharan Africa. Current estimates suggest that nearly half of the global population is at risk of malaria and there are annually approximately 250 million cases resulting in a million deaths [1]. In addition, *An. gambiae *vectors o'nyong-nyong virus (ONNV), a single-stranded (+) RNA virus (Togaviridae; Alphavirus) [2-4]. Symptoms of ONNV infection in humans include rash, fever and polyarthritis often resulting in high morbidity rates during epidemics [5,6].

Although most arthropod-borne viruses (arboviruses) are vectored by culicine mosquitoes, ONNV is primarily vectored by the anopheline mosquitoes *An. gambiae *and *An. funestus *[7]. In spite of the unusual vector specificity, ONNV shares a common host cell entry mechanism with many other arboviruses. Endocytosis and subsequent fusion with the host's membrane in the endosome are exploited by ONNV to infect host cells [8]. Alphaviruses, including ONNV, Sindbis virus, and Chikungunya virus have class II fusion proteins such as E glycoproteins that mediate membrane fusion between virus and host cells during virus entry [8,9]. Class II E glycoproteins mainly consist of beta sheet-folded domains while class I E proteins are α-helices [10,11]. Since membrane fusion is one of the protein maturation processes mediated by molecular chaperones, such as the HSP70 family, it is possible that HSP70 may enhance or suppress maturation of viral proteins [12-14].

Members of the HSP70 family contain three conserved domains: an ATPase domain at the N-terminus, a peptide binding domain, and a GP-rich region at the C-terminus that contains an EEVD motif [15-17]. HSP70, a molecular chaperone, changes its conformation in an ATP dependent manner to mediate proper target protein folding, degradation and translocation [18,19]. The carboxy-terminal EEVD motif is a unique feature of cytosolic heat shock proteins that is recognized by chaperone cofactors to initiate chaperone activity [20-22]. The heat shock cognate protein 70 (HSC70) is a constitutively expressed member of the HSP70 family and functions as a molecular chaperone under normal cellular conditions. However, the expression of the HSC70 gene may be increased in response to environmental and physiological stress [19].

The *An. gambiae *HSC70B is an ortholog of *Drosophila melanogaster *Hsc70-4 [23]. cDNA microarray studies demonstrated that HSC70B is upregulated during ONNV infection in adult *An. gambiae*, suggesting an important role during virus infection [24]. The functional importance of HSC70B upregulation in ONNV-infected female *An. gambiae *was further elucidated by RNAi gene silencing of the *hsc70B *transcript [23]. Reduction of the *hsc70B *transcript by RNAi silencing enhanced ONNV replication *in vivo*. Likewise, enhanced ONNV replication in HSC70B-knockdown mosquitoes suggests that HSC70 proteins play an important role in arbovirus suppression and maintaining homeostasis during infection [23].

To further elucidate the transcriptional regulation of the *hsc70B *locus in response to viral infection, we characterized the 5' upstream region of the *hsc70B *coding sequence *ex vivo *using cell culture and luciferase reporter systems. Herein, we report the identification of a regulatory region essential for *hsc70B *transcription. Furthermore, the kinetic properties of *hsc70B *transcription during arbovirus infections were examined with ONNV (Togaviridae; Alphavirus), West Nile virus (Flaviviridae; Flavivirus) and La Crosse virus (Bunyaviridae; Orthobunyavirus). The results showed that the *hsc70B *promoter region was responsive to all three arboviruses. Induction of *hsc70B *transcription by three taxonomically different arboviruses suggests that the HSC70B protein may be expressed to cope with cellular stress imposed during infection. The biological implications of these data are discussed.

## Results

### Sequence analysis of the 5' upstream of *hsc70B*

**Figure 1:**
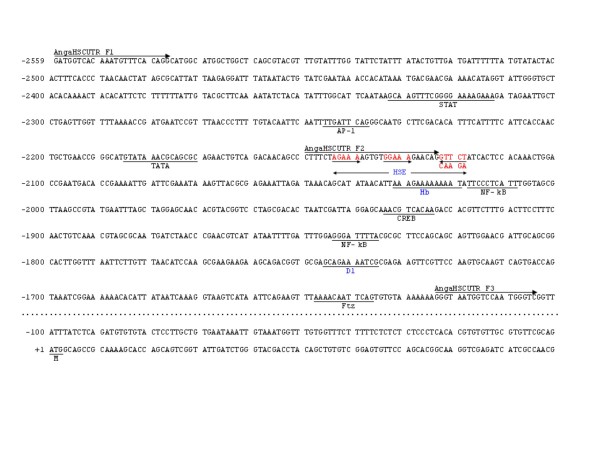
**Nucleotide sequence of the *hsc70B *promoter region**. Putative binding sites for transcription factors are underlined. The binding sites were evaluated *in silico *by both the TFSEARCH and AliBaba2.1 program. Transcription factors predicted by both programs are marked blue. The consensus sequence of HSE (5'-NGAAN-3') is marked red. The position +1 denotes the first base of the putative starting codon ATG.

Transcription factor binding elements along the 5'-upstream sequence of the *hsc70B *gene (2559 bp) were analyzed *in silico*. The binding sites identified by both the TFSEARCH and AliBaba2.1 programs are shown in Figure 1. In addition to core promoter sequences (*e.g*., TATA and CAT boxes), putative binding sites for heat shock proteins such as HSE-bind and heat shock transcription factor (HSF) were also identified. Putative binding sites for NF-κB, Dl, c-AMP response element binding protein (CREB), signal transducers and activators of transcription protein (STAT), and fushi-tarazu (Ftz) factors were also identified.

### Deletion analysis of the *hsc70B *promoter

**Figure 2:**
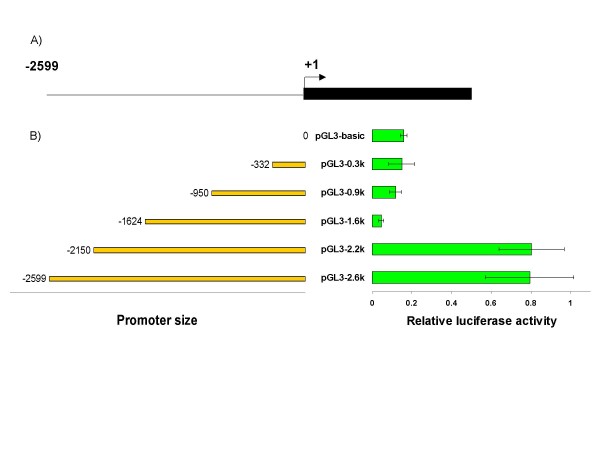
**Deletion Analysis of the *hsc70B *promoter**. (A) The solid black line represents the full length of the promoter where positions -2599 and +1 denote the 5' end of the *hsc70B *promoter and the putative starting codon ATG, respectively. (B) The bars on the left represent the lengths of the 5' upstream region that were generated by PCR. The bars on the right represent relative firefly luciferase activities (mean ± SD) that were normalized by the *Renilla *luciferase activity. The relative luciferase activity indicates the promoter activity of the 5' upstream deletion constructs of *hsc70B*. The promoter activities of constructs less than 2.2 kb were significantly lower than the 2.6 kb full length construct.

To identify the critical elements required for transcription, various deletions of the 5' upstream region of the *hsc70B *locus were generated and ligated into the pGL3-Basic vector. The promoter activities of the different deletion constructs were compared to that of the full-length construct (2.6 kb). The full length promoter pGL3-2.6k contains 2599 bp of the 5' upstream region (-2599 to -1); +1 denotes the first base of the starting codon (Figure 2A). Deletions of 449 bp, 975 bp, 1649 bp and 2267 bp from the 5' end of the full length promoter produced pGL3-2.2k, pGL3-1.6k, pGL3-0.9k and pGL3-0.3k, respectively. The promoter activity of these deletion constructs was measured by firefly luciferase expression and normalized by the *Renilla *luciferase expression. Both pGL3-2.6k and pGL3-2.2k constructs had luciferase expression levels 5-fold higher than that of the pGL3-Basic control. The luciferase expression levels of pGL3-0.3k, pGL3-0.9k and pGL3-1.6k did not differ from that of the control (Figure 2B). These data suggest that elements critical for the transcription of *hsc70B *reside in the 526 bp region between 2.2 and 1.6 kb upstream of the starting codon.

### Effect of ONNV infection on the pGL3-2.6k and pGL3-2.2k *hsc70B *promoter plasmid constructs

**Figure 3:**
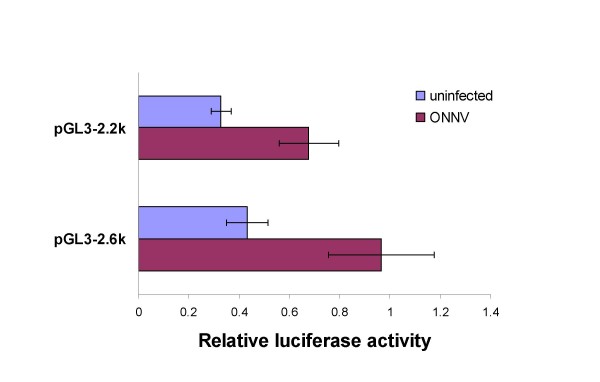
**Induction of the *hsc70B *promoter in transfected Vero cells by ONNV infection**. The constructs containing the 2.6 kb and 2.2 kb-long 5' upstream regions were evaluated for *hsc70B *promoter activity during ONNV infection (MOI = 0.001). The luciferase activity was measured at 60 h post ONNV infection when CPE became apparent. The *hsc70B *promoter activity, as measured by relative luciferase activity (mean ± SD), was significantly elevated in both constructs when compared to uninfected controls (2.2 kb uninfected vs. infected: P < 0.01, t = 4.702, df = 6; 2.6 kb uninfected vs. infected: P < 0.01, t = 5.681, df = 6).

To determine if differences between the promoter activities of the pGL3-2.6k and pGL3-2.2k constructs occurred during arbovirus infection, the constructs were initially evaluated in the context of ONNV infection. Transfected with either the pGL3-2.6k or pGL3-2.2k plasmids, Vero cells were subsequently infected with ONNV (MOI = 0.001). The cells were harvested after cytopathic effects (CPE) were confirmed at 60 hpi. ONNV infection significantly increased the *hsc70B *promoter activity (Figure 3). The luciferase activity of both pGL3-2.6k and pGL3-2.2k constructs in ONNV-infected Vero cells was ~2-fold higher than uninfected Vero cells.

### Effect of arbovirus infection on the *hsc70B *promoter activity

**Figure 4:**
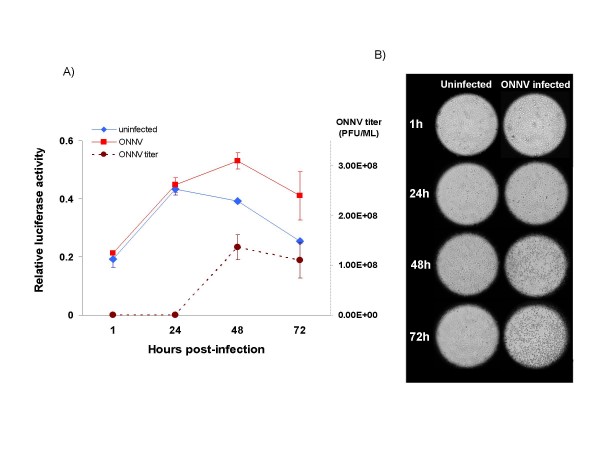
***hsc70B *promoter activity (mean ± SD) time course experiments during ONNV infection**. (A) The *hsc70B *promoter activity, as measured by luciferase activity, was significantly higher at 48 h (P < 0.01, t = 8.53, df = 4) and 72 hpi (P < 0.01, t = 27.34, df = 4) in the ONNV infected samples. ONNV titers were also markedly elevated at 48 and 72 hpi. The induction of the HSC70B promoter corresponds to viral titer. (B) ONNV cytopathic effects in Vero cells; CPE are clearly evident at 48 and 72 hpi.

Based on the previous results, the pGL3-2.2k construct was used to assay the effect of arbovirus infection on the *hsc70B *promoter. A time course experiment with ONNV (MOI = 0.001) in Vero cells transfected with the pGL3-2.2k construct demonstrated increases in *hsc70B *promoter activities at 48 and 72 hpi. However, at earlier time points the *hsc70B *promoter activity was comparable to that of the uninfected control (Figure 4A). This enhanced *hsc70B *promoter activity in ONNV-infected cells appeared to occur with increasing ONNV titers at 48 and 72 hpi. The titers were 1.5 × 10^2^, 3 × 10^5^, 1.4 × 10^8^, and 1.1 × 10^8 ^plaque forming units (pfu)/mL at 1, 24, 48 and 72 hpi, respectively (Figure 4A). Furthermore, CPE in ONNV-infected Vero cells became evident at 48 hpi, corresponding with the elevated viral titers at later time points (Figure 4B). The *hsc70B *promoter activity in ONNV infected Vero cells was 1.4 and 1.6-fold higher at 48 hpi and 72 hpi, respectively, than that in uninfected Vero cells (Figure 4A).

**Figure 5:**
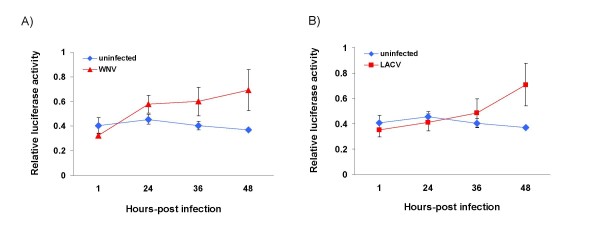
**Increased *hsc70B *promoter activities (mean ± SD) in Vero cells during West Nile virus (A) and La Crosse virus (B) infection**. The *hsc70B *promoter activity, as measured by luciferase activity, is higher in infected cells at 36 and 48 hpi.

To determine if the observed transcriptional activation of *hsc70B *was virus specific, two taxonomically distinct arboviruses were chosen for additional time course experiments. Vero cells transfected with pGL3-2.2k constructs were infected with WNV or LACV at MOI = 0.01. The infected cells were harvested at 1, 24, 36 and 48 hpi. Infection with either virus also significantly increased the *hsc70B *promoter activity as determined by the luciferase assay (Figure 5).

## Discussion

Repression of ONNV replication by the HSC70B protein was previously shown in *An. gambiae *[23,24]. Of particular interest in this result is the transcriptional regulation of *hsc70B *expression in response to ONNV infection in *An. gambiae*. To map and characterize the promoter activity of *hsc70B*, the upstream region up to 2599 bp from the putative starting codon of *hsc70B *was subjected to a luciferase reporter assay. Initially, the 2599 bp upstream sequence of the *hsc70B *showed a promoter activity (Figure 2). Subsequent deletion analysis of this region revealed that the regulatory elements critical for *hsc70B *transcription reside between 2150 ~ 1615 bp upstream of the *hsc70B *starting codon (Figure 2). Deletion of this 535 bp region abolished the promoter activity of *hsc70B*. This regulatory region contains several binding sites for transcription factors such as HSE-bind, CRE, NF-κB, dorsal, and Ftz (Figure 1). HSE is a binding site for heat shock transcription factors that are activated in response to environmental and physical stresses such as heat shock and microbial infection [25,26]. In *hsc70B*, there is one putative HSE consisting of a block of three repeats of a 5-bp sequence, 5'-nGAAn-3'. Although the number of HSE blocks can vary among different HSPs, the 5-bp HSE repeat is highly conserved in the regulatory region of various heat shock proteins such as *hsp70*, *hsp83*, and *hsp27 *in *Drosophila *[27]. The second and third repeat in the HSE block of *An. gambiae hsc70B *has a tail-to-tail (5'nTTCnnGAAn3') arrangement with 6-bp gaps between them (Figure 1). In *Drosophila *HSPs, there are 5 or less gaps, if any, between the 5-bp repeats [27]. It will thus be interesting to learn how the additional gap in *An. gambiae hsc70B *regulates *hsc70B *expression.

CRE is a response element for phosphorylated CREB (c-AMP response element-binding protein) which regulates transcription of genes. CREB is involved in human hsp90 gene expression which is constitutively expressed [28]. Thus, CRE may be a key element to induce basic transcription of *An. gambiae hsc70B *gene as it is also a constitutively expressed member of HSPs. NF-κB is a transcription factor which responds to stresses including viral infection [29]. Transcription of NF-κB was shown to be increased by downregulation of HSC70B protein in rat pancreatic acinar AR42J cells [30]. Ftz is a transcription factor that was originally isolated in *Drosophila*. It has many orthologs in various species and is involved in *fushi tarazu *gene expression which functions in embryonic segmentation in *Drosophila *and sex determination in zebrafish [31,32]. Further biochemical and molecular characterization using electrophoretic mobility shift assays (EMSA) and DNase I protection assay should elucidate key elements that transcriptionally regulate *An. gambiae hsc70B *expression in response to ONNV infection. These assays will further improve our understanding of transcriptional regulation of *hsc70B*, and facilitate the identification of transcriptional factors and co-factors in the signal transduction pathway of *hsc70B *expression.

ONNV was used to infect Vero cells to examine the effects on *hsc70B *promoter activity. The different lengths, 2150 bp and 2599 bp, of the 5' upstream sequences were tested because these two constructs contain the regulatory sequence for the basic transcription of *hsc70B*. Both 2150 bp and 2599 bp upstream genomic fragments responded to ONNV infection and the promoter activities of both constructs increased during ONNV infection (Figure 3). When Vero cells were transfected with either pGL3-2.6k or pGL3-2.2k reporter plasmid, the promoter activity in the reporter plasmids was about 2-fold higher in infected cells than the uninfected control (Figure 3). This suggests that induction of *An. gambiae hsc70B *gene, leading to expression of the HSC70B protein, results from virus infection. Therefore, it is reasonable to speculate that cellular signals are transduced to the regulatory region of the *hsc70B *locus in *An. gambiae*.

The 2150-bp 5' upstream sequence was used to further investigate the effects of ONNV infection on the *hsc70B *promoter activity at different time points after infection. The promoter activity of *hsc70B *was significantly higher in infected cells at later time points (48 and 72 hpi) than earlier points (1 and 24 hpi) (Figure 4A). The elevated *hsc70B *promoter activity corresponded with increasing viral titers at 48 and 72 hpi because plaque assays of the cell culture media showed higher ONNV titers at these later time points (Figure 4A). These plaque assay data were further evaluated by observing CPE in ONNV-infected Vero cells. CPE became apparent at 48 and 72 hpi in Vero cells while uninfected control cells did not show cell lysis (Figure 4B). The appearance of CPE in ONNV-infected Vero cells corresponded to higher ONNV titers at 48 and 72 hpi. It can be thus inferred that induction of *hsc70B *transcription may be triggered in response to cellular stresses burdened by rapidly replicating viruses. In cells at immediate or early infection stages, *hsc70B *expression may not be activated.

The inducibility of the *hsc70B *promoter was also examined using two additional arboviruses, WNV (Flaviviridae) and LACV (Bunyaviridae). Like ONNV, both WNV and LACV were also able to upregulate the transcription activity of *hsc70B *during infection (Figure 5). Due to more rapid kinetics of replication, both WNV and LACV caused the *hsc70B *promoter activity to rise earlier than ONNV. For example, WNV-infected Vero cells started to show transcriptional induction as early as 24 hpi. Transcriptional activation of *hsc70B *by three different arboviruses suggests that upregulation of *hsc70B *expression indeed results from cellular stresses caused by virus infection in host cells. In addition, activation of the *hsc70B *promoter by virus infection was recently shown in shrimp (*Penaeus monodon*) [33]. Using a luciferase reporter in Sf21 cells, Chuang *et al*. (2007) demonstrated 5.5-fold induction of the shrimp *hsc70B *promoter when the Sf21 cells were infected with *Autographa californica *multiple nuclear polyhedrosis virus (Ac*M*NPV; MOI = 0.1). Therefore, it appears that induction of *hsc70B *expression may be a general cellular response of host cells to virus infection.

## Conclusion

We previously reported that the transcriptional activation of *hsc70B *in ONNV-infected *An. gambiae *renders the mosquito an ability to repress ONNV replication [23,24]. These *in vivo *findings and our current *ex vivo *characterization of the *hsc70B *regulatory region unequivocally indicate that the induction of HSC70B may be a mosquito innate immune response against virus infection. To support this hypothesis, mosquito cells (e.g., C6/36 cells from *Ae. albopictus*) do not show any CPE during arbovirus infection while mammalian cells including Vero cells display prominent CPE and subsequent cell lysis due to overreplication of viruses. Evolutionally, mosquitoes may have acquired the ability to maintain viral titers below a certain threshold, below which mosquitoes may serve as arboviral vectors without pathogenesis from viral infections. Interestingly, a potent antiviral drug, prostaglandin A, showed antiviral effects against Sendai or Sindbis virus through induction of HSP70 proteins in AGMK cells (African green monkey kidney) or Vero cells, respectively [34,35]. Therefore, comparative studies on HSP expression in response to viral infection between mosquito and mammalian cells will provide a deeper insight into innate immune responses to viral infection between mosquito vectors and mammalian hosts.

## Methods

### Construction of *An. gambiae hsc70B *promoter-luciferase reporter gene

The 2599 bp 5' region upstream of the putative starting codon of the *hsc70B *gene was amplified from BAC clone 132E18  by a PCR method using Phusion High-Fidelity DNA polymerase (NEB, MA). The primers used were as follows: AngaHsc_F1, 5'-CCCGAGCTCGATGGTCACAAATGTTTCACAGG-3' and AngaHsc_R, 5'-CCGCTCGAGCTGCGAACACGCAACACAC-3' with a *SacI *or an *XhoI *recognition site (underlined) incorporated at the 5' end of the primers, respectively. The PCR conditions were as follows: 98°C for 30 sec, followed by 30 cycles of denaturation at 98°C for 10 sec, annealing at 68°C for 30 sec and extension at 72°C for 80 sec, a final extension at 72°C was performed for 10 min. The amplified DNA fragment was double-digested with *SacI *and *XhoI *and subcloned into the promoterless pGL3-Basic vector (Promega) predigested with *SacI *and *XhoI *to construct pGL3-2.6k. Serial deletions of the 5'-flanking region of the *hsc70B *gene were also prepared from pGL3-2.6k using a PCR method with the primers listed in table 1.

**Table 1:** Primer sequences used to construct pGL3-*hsc70B *plasmids

Primers	Primer sequence (5' to 3')	Position	Usage
AngaHsc_F1	CCCGAGCTCGATGGTCACAAATGTTTCACAGG	-2599	Forward primer to construct pGL3-2.6k
AngaHsc_F2	CCCGAGCTCCTTTCTAGAAAAGTGTGGAAAGAACAG	-2150	Forward primer to construct pGL3-2.2k
AngaHsc_F3	CCCGAGCTCGGGTAATGGTCCAATGGGTC	-1624	Forward primer to construct pGL3-1.6k
AngaHsc_F4	CCCGAGCTCTGTGAAATGTCCTAATTTTTTGCC	-950	Forward primer to construct pGL3-0.9k
AngaHsc_F5	CCCGAGCTCGCATCATGCGTTAGGTCTCAG	-332	Forward primer to construct pGL3-0.3k
AngaHsc_R	CCGCTCGAGCTGCGAACACGCAACACAC	-1	Reverse primer to construct all plasmids

Restriction enzyme recognition sites are underlined. *SacI*: GAGCTC; *XhoI*: CTCGAG

### Analysis of 5' upstream sequence of *hsc70B*

Putative binding sites for transcription factors in the 5' upstream region of *hsc70B *were predicted *in silico *using the TFSEARCH [36,37] and AliBaba2.1 [38] programs.

### Transfection and luciferase activity assay of the *hsc70B *promoter activity in Vero cells

Transfection experiments were performed in 24-well plates using the Lipofectamine reagent according to the manufacturer's instructions (Invitrogen, CA). Briefly, Vero cells (ATCC: CCL-81) were seeded and incubated at 37°C with 5% CO_2 _in Dulbecco's Modified Eagle Medium (DMEM) for 24 h prior to transfection at a density of 0.5 × 10^5 ^cells/well. When the cells reached ~80% confluency, the culture media was removed and 200 μl of fresh DMEM without antibiotics or fetal bovine serum (FBS) was added. The cells were then co-transfected with 400 ng of pGL3 firefly (*Photinus pyralis*) luciferase constructs containing varying lengths of the *hsc70B *upstream region (*e.g*., pGL3-2.6k, pGL3-2.2k, pGL3-1.6k, pGL3-0.9k, pGL3-0.3k, or pGL3-Basic) and 0.05 ng of a pRL-cmv *Renilla reniformis *luciferase construct. The pRL-cmv construct was used as an internal control, in which the *Renilla *luciferase expression is driven by the cytomegalovirus promoter (cmv). Because the pGL3-Basic is a promoterless reporter plasmid containing only the coding sequence of firefly luciferase it served as a background control. At 3 h post transfection, the transfection mixture was replaced with a complete medium including 100 U/mL Penicillin-Streptomycin, and 10% FBS. Cells were harvested at pre-defined time points post transfection. The luciferase activities were measured by the Dual-Luciferase Reporter Assay System (Promega, WI) according to manufacturer's instructions. Quantification of the luminescent signals was performed using a Synergy HT microplate reader (BioTek, USA). In order to account for heterogeneous transfection efficiencies and cell viabilities among different samples, the firefly luminescence values were normalized as a ratio of the *Renilla *luminescence values. A minimum of three biological replicates were included for the time course experiments with ONNV. For time course experiments with WNV and LACV, the mean values and standard deviations were calculated from four biological replicates out of six replicates. The largest and the smallest values from these replicates were excluded from the analysis.

### Viruses

The SG650 strain of ONNV has previously been described [23]. The WNV isolate (LA-11-2005) was isolated by BDB from the brain tissue of a blue jay (*Cyanocitta cristata*) found in New Iberia, LA during 2005. A cloacal swab from the bird tested positive for WNV by the Rapid Analyte Measurement Platform (RAMP, Adapco, Inc.). Subsequent nucleic acid amplification and sequencing of the PreM-Envelope region of the isolate confirmed the RAMP identification (GenBank Accession Number DQ646699). The virus was isolated in Vero cells and had not been further passaged. The LACV (78-V-13193) was obtained from the World Reference Center for Arboviruses at the University of Texas Medical Branch, Galveston, TX. The virus had been passed once in suckling mouse brain and twice in Vero cells.

### Virus infection

To determine the effect of viral infection on the promoter activity of *hsc70B*, Vero cells cotransfected with pGL3-2.6k or pGL3-2.2k and pRL-cmv were infected with ONNV, WNV or LACV 12 h post transfection. For ONNV, confluent monolayers of Vero cells were infected at an MOI (multiplicity of infection) of 0.001. The infected cells were harvested at predetermined time points (*e.g*., 1, 24, 48 and 72 h post-infection) during time course experiments. Otherwise, the cells were harvested at 60 h post infection when CPE were evident. For the WNV and LACV time course experiments, confluent monolayers of Vero cells were infected at an MOI of 0.01 and the infected cells were harvested at 1, 24, 36 and 48 hpi. Viral titers were determined by a standard plaque assay in Vero cells [39].

## Competing interests

The authors declare that they have no competing interests.

## Authors' contributions

SK performed the experiments, analyzed the data, and drafted the manuscript. CS contributed to the cloning of the *hsc70B *locus and reviewed the manuscript. BDB conducted cell culture and viral plaque assays and reviewed the manuscript. FHC initiated the project and provided materials and a critical review of the manuscript. YSH provided overall direction and conducted experimental design, data analysis and wrote the manuscript. All authors read and approved the final manuscript.
